# Risk Assessment of Heavy Metals Contamination in Paddy Soil, Plants, and Grains (*Oryza sativa* L.) at the East Coast of India

**DOI:** 10.1155/2014/545473

**Published:** 2014-06-03

**Authors:** Deepmala Satpathy, M. Vikram Reddy, Soumya Prakash Dhal

**Affiliations:** ^1^Department of Ecology and Environmental Sciences, Pondicherry University, Pondicherry 605 014, India; ^2^Department of Physics, Pondicherry University, Pondicherry 605 014, India

## Abstract

Heavy metals known to be accumulated in plants adversely affect human health. This study aims to assess the effects of agrochemicals especially chemical fertilizers applied in paddy fields, which release potential toxic heavy metals into soil. Those heavy metals get accumulated in different parts of paddy plant (*Oryza sativa* L.) including the grains. Concentrations of nonessential toxic heavy metals (Cd, Cr, and Pb) and the micronutrients (Cu, Mn, and Zn) were measured in the paddy field soil and plant parts. Mn and Cd are found to be accumulated more in shoot than in root. The metal transfer factors from soil to rice plant were significant for Pb, Cd, Cu, Cr, Mn, and Zn. The ranking order of bioaccumulation factor (BAF) for heavy metals was Zn > Mn > Cd > Cu > Cr > Pb indicating that the accumulation of micronutrients was more than that of nonessential toxic heavy metals. The concentrations of heavy metals were found to be higher in paddy field soils than that of the nearby control soil but below permissible limits. The higher Health Index (HI) values of rice consuming adults (1.561) and children (1.360) suggest their adverse health effects in the near future.

## 1. Introduction

Heavy metals from natural and anthropogenic sources accumulate in soil and plants and as a consequence represent important environmental contamination problems. Nevertheless, food safety issues and adverse health risks make this one of the most serious environmental issues [1]. Soils are considered to be an excellent media to monitor and assess heavy metal pollution because anthropogenic heavy metals are usually deposited in the top soils [2]. Heavy metal contaminated soil adversely affects the whole ecosystem when these toxic heavy metals migrate into groundwater or are taken up by flora and fauna, which may result in great threat to ecosystems due to translocation and bioaccumulation [3]. Heavy metals are potentially toxic to crop plants, animals, and human beings when the contaminated soils are used for crop production [4]. Environmental contamination of the biosphere with heavy metals due to intensive agricultural and other anthropogenic activities poses serious problems for safe use of agricultural land [5]. Contemporary agriculture with indiscriminate use of agrochemicals such as fertilizers and pesticides, along with mechanical cultivation, for higher crop productivity contaminates agriculture soils with potentially nonessential and essential heavy metals [6, 7]. Human health is directly affected through intake of crops grown in polluted soils. There is clear evidence that human renal dysfunction is related with contamination of rice with Cd in subsistence farms in Asia [8]. Indeed, in Asia, rice has been identified as one of the major sources of Cd and Pb for human beings [9–11]. In Japan, rice was found to be the main source of Cd contamination in human beings [12].

It has also been reported that crop plants have different abilities to absorb and accumulate heavy metals in their body parts and that there is a broad difference in metal uptake and translocation between plant species and even between cultivars of the same plant species [13–20]. Plants absorb heavy metals from the soil, and the surface 25 cm zone of soil is mostly affected by such pollutants resulting from anthropogenic activities. Heavy metals are adsorbed and accumulated in this soil layer probably due to relatively high organic matter. The plant parts of interest for direct transfer of heavy metals to human body are the edible parts such as the rice grain, which may consequently become a threat to human health. Nevertheless, heavy metals in the environment, consequently, are of immense concern, because of their persistence nature, bioaccumulation, and biomagnification characters causing ecotoxicity to plants, animals, and human beings [21].

The micronutrients such as Zn, Mn, and Cu are required in small but critical concentrations for both plants and animals, and these have vital role in physical growth and development of crop plants such as paddy. The deficiency of Zn in soil casts a conspicuous adverse effect, with stunted growth of crop plants like paddy and groundnut [22, 23] reducing the overall productivity. Generally, the monitoring and assessment of total heavy metal concentrations in agricultural soils are required to evaluate the potential risk of paddy soils contaminated due to toxic heavy metals—Cd, As, and Pb [7, 24]. Heavy metals are known to accumulate in living organisms [25]. There is a tendency of plants to take up heavy metals that may subsequently transfer into the food chain. Use of polluted soil or water for crop cultivation mainly results in decrease of overall productivity and contaminates food grains and vegetables, which adversely affect human health too [26]. A number of reports on concentrations of toxic metal such as Cd, and Pb in rice and paddy soils in Japan, China, and Indonesia are available [27–31]. However, such studies are very few in India, with little information on toxic heavy metal contamination of paddy fields and risk assessment [32], though rice is the most important staple food for Indian people. The objectives of the present study, therefore, are primarily risk assessment of potential toxic and nonessential heavy metals—Cd, As, Pb, and Cr, in the surface soil of paddy fields at the predominantly paddy-cultivated area nearby Kalpakkam (Tamil Nadu). Concentrations of the toxic heavy metals were assessed in soil, root, shoot, and grains of paddy crop to assess the bioaccumulation factor and transfer factor. Risk assessment was made assessing the potential risk factor for the local residents consuming rice, the staple food.

## 2. Materials and Methods

### 2.1. Study Site

**Figure 1:**
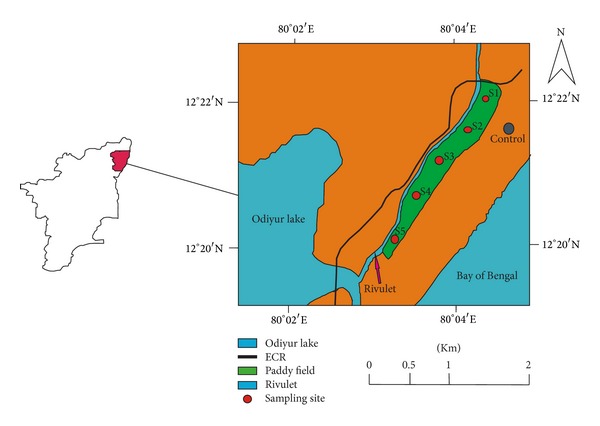
Location of sampling area.

The study site is located at about 30 km away from Pondicherry towards east on the East Coast Road (ECR) at Swarnabhoomi, near Kalpakkam in Tamil Nadu, India (12°22′21.5′′ and 12°21′32.4′′ N to 80°04′36.6′′ and 80°04′ 04.2′′ E0 (Figure 1)). The agricultural watershed is of about 50 ha, and it is transected at one end by the ECR. About 30 ha of the watershed comprising of paddy fields applied with chemical fertilizers and pesticides, shed their water into adjacent rivulet which finally joins a small lagoon located towards south-west. About 20 ha of the paddy fields is on the same plane as that of the rivulet, from where the water is taken for irrigation of the fields. Sampling of the soil and rice plants with grains was carried out during crop season (winter 2012) in order to investigate the concentrations and spatial distribution of potentially toxic heavy metals such as Pb, Cd, Cu, Cr, Mn, and Zn originated from the agricultural activities of the agriculture watershed. The study site and sampling locations of the study site are shown in Figure 1.

#### 2.1.1. Sampling of Soil, Rice Plants, and Grain and Sample Preparation

Soil samples were collected from six different sites of the paddy fields. Out of these one was the control site where no crop cultivation was done and other five sites (S-1, S-2, S-3, S-4, and S-5) were selected from paddy fields. At each sampling site, a composite of five soil samples was collected separately by a random selection, from each field, from surface (0–15 cm soil layer) with a small core sampler and mixed to make one composite sample. Samples were collected from centre of the fields in order to avoid the edge effect. Each soil sample of about 500 mg was collected from the 0–15 cm layer, which represented the plough layer. Rice plant samples were collected from the corresponding soil sampling site of the paddy field for computing correlations between heavy metal concentrations of soil and plant. All soil and rice plants along with grain samples were kept in clean polyethylene bags and brought to the laboratory for analyses. During plant sampling, it was ensured that plant samples were of the same physiological age and identical size. Paddy crop plants were collected and washed thoroughly with deionized water. Paddy plant was cut and separated into root, shoot, and grain subsamples. All subsamples were oven-dried at 60°C for 24 h, and the dried samples were weighed, then pulverized, and stored in Petri dishes. The soil samples were air-dried at room temperature for several days, then pulverized, and sieved through a 01 mm stainless steel mesh. Rice grain samples were washed with deionized water and hulls were removed. The rice grain samples without hull were oven-dried at 70°C for 72 h and then ground with an agate mortar to fine powder.

### 2.2. Sample Analysis

Soil pH and conductivity were determined by using a digital pH meter. For heavy metal analysis, one gm of soil and 1 g of rice grain samples were digested after adding 15 mL of triacid mixture (HNO_3_, H_2_SO_4_, and HClO_4_ in a 5 : 1 : 1 ratio) with three replicates at 80°C until a transparent solution was obtained [33]. After cooling, the digested sample was diluted up to 30 mL with 2% HNO_3_ and concentrations of Pb, Cd, Cu, Cr, Mn, and Zn were determined by AAS (GBC make—Model Avanta PM).

For plant samples, 1 g of dried sample was digested with HNO_3_ and HClO_4_ in a 5 : 1 ratio until a transparent solution was obtained, and the plant digests were filtered and diluted to 30 mL, with distilled water (Reddy et al.) [32]. The filtrates of plant were then assessed by using atomic absorption spectroscopy (AAS; GBC make—Model Avanta PM) for analysis of Pb, Cd, Cu, Cr, Mn, and Zn. The AAS value of blank (without sample) of each metal was deducted from the sample value for final calculations [26]. All the analyses were done with three replications.

### 2.3. Bioaccumulation Factor (BAF)

The BAF (bioaccumulation factor, the ratio of the concentration of the element in the grain to that in the corresponding soil) was calculated for each rice sample to quantify the bioaccumulation effect of rice on the uptake of heavy metals from the soils [34]. The BAF was computed as

(1) $$\begin{array}{c} \text{BAF}=\frac{\text{Cr}}{\text{Cs}}, \end{array}$$

where Cr and Cs represent the heavy metal concentrations in rice grain and soils, respectively.

### 2.4. Translocation Factor (TF) and Enrichment Factor (EF)

Translocation factor (TF) or mobilization ratio [35, 36] was calculated to determine relative translocation of metals from soil to other parts (root, shoot, or grain) of the plant species as follows:

(2) TF=Concentration of metal in plant tissueConcentration of metal in corresponding soil or root or shoot.

The enrichment factor (EF) has been calculated to derive the degree of soil contamination and heavy metal accumulation in soil and in plants growing on contaminated site with respect to soil and plants growing on uncontaminated soil [37] as follows:

(3) $$\begin{array}{c} \text{EF}=\frac{\text{Concentration}\;\text{of}\;\text{metals}\;\text{in}\;\text{soil}\;\text{at}\;\text{contaminated}\;\text{site}}{\text{Concentration}\;\text{of}\;\text{metals}\;\text{in}\;\text{soil}\;\text{at}\;\text{uncontaminated}\;\text{site}}. \end{array}$$

### 2.5. Risk Assessment

The Health Risk Index (HRI) was calculated as the ratio of estimated exposure of rice and oral reference dose (ORD) [7]. ORDs were 2 × 10^−2^, 1 × 10^−3^, and 4 × 10^−2^ and 1.5 mg/kg/day for Pb, Cd, Cu, Cr, and Zn, respectively [38, 39]. Estimated exposure is obtained by dividing the daily intake (DI) of heavy metals by their safe limits. An index value >1 is considered unsafe for human health [39]. DI was calculated by the following equation:

(4) $$\begin{array}{c} \text{DI}=\frac{\text{C}\times \text{Con}\times \text{EF}\times \text{ED}}{\text{Bw}\times \text{AT}}, \end{array}$$

where C (in milligrams per kilogram) is the concentration of heavy metals in the rice, Con (in grams per person per day) is the daily average consumption of rice in the region, Bw (in kilograms per person) represents body weight, EF is exposure frequency (365 days/year), ED is exposure duration (70 years, equivalent to the average lifespan), and AT is average time (365 days/year number of exposure years, assuming 70 years in this study). The average daily rice intake of adults and children was considered to be 389.2 and 198.4 g/person/day, respectively [40]. Average adult and child body weights were taken to be 55.9 and 32.7 kg, respectively, as used in many previous studies [7, 40–42]. The health risk for adult and children is considered separately since the contact pathway with each exposure way changes with age. There may be a certain amount of discrepancy in health risk between age groups and locality of inhabitants (Wang et al. [41]). Harrison and Chirgawi [43] reported that exposure of two or more pollutants may result in additive or interactive effects. Wang et al. [41], Chien et al. [44], Zheng et al. [40], and Hang et al. [7] have studied hazard index for different diets. Assuming the additive effect here HRI can, therefore, be summed across the constituents to calculate the HI for a specific receptor (e.g., diet) combination. The HI is calculated to evaluate the potential risk of adverse health effects from a mixture of chemical constituents in rice. The HI was calculated through daily average consumption of rice for a human being (adults and children) and is as follows:

(5) $$\begin{array}{c} \text{HI}=\overset{n}{\underset{i=1}{\sum }}\text{HRI}. \end{array}$$

### 2.6. Data Analysis

Arithmetical means ± standard deviation (SD; *n* = 3) were used to assess the contamination levels of heavy metals in soils, root, shoot, and grains. Coefficient correlation analysis was done to find out the heavy metals characteristics in agricultural field soil and grain samples.

## 3. Results and Discussion

### 3.1. Heavy Metal Concentration in Soil

It was found that, in the essential heavy metals in the paddy soil, Mn concentration ranged from 12.5 to 53.9  *μ*g g^−1^, Zn concentration ranged from 3.8 to 33.8 *μ*g g^−1^, Cu concentration ranged from 0.03 to 2.9 *μ*g g^−1^ in the paddy field soils, and in the concentrations of nonessential toxic metals, Pb ranged from 5.3 to 19.8 *μ*g g^−1^, Cr ranged from 1.3 to 7.8 *μ*g g^−1^, and Cd from 0.02 to 0.6 *μ*g g^−1^ (Table 1). Among these metals, Cd and Cr are highly toxic, while Pb is moderately toxic and Zn, Mn, and Cu are essential elements and micronutrients [45]. The ranking order of occurrence of the heavy metals in the paddy field soils was Mn > Zn > Pb > Cr > Cu > Cd indicating that Mn followed by Zn was in the maximum concentrations and Cd was in minimum concentration. Concentrations of the heavy metals are higher in the paddy field soils compared to that of nearby control field soil. However, the ranking order of concentration of the metals is different from that of the paddy soil (Cd > Mn > Zn > Cu > Pb) of a predominantly paddy cultivated area at Bahour near Puducherry, with Cd in maximum concentration and Pb in minimum concentration [32]. The concentrations of Pb and Cd were higher in S-5 (19.8 ± 1.3 *μ*g g^−1^ and 0.6 ± 0.04 *μ*g g^−1^), respectively, followed by S-4 (17.3 ± 0.9 *μ*g g^−1^ and 0.5 ± 0.02 *μ*g g^−1^), respectively, the concentrations of Pb were 14.9 ± 0.3 *μ*g g^−1^ at S-2 and 11.7 ± 0.6 *μ*g g^−1^ at S-1, and Cd concentration was (0.4 ± 0.007 *μ*g g^−1^) at S-1, showing the ranking order of Pb concentration in paddy soil, S-5 > S-4 > S-3 > S-2 > S-1. Concentration of Cd was higher in S-5 followed by S-4, S-3, and S-2 (0.2 ± 0.03 *μ*g g^−1^) showing the ranking order of S-5 > S-4 > S-3 > S-1 > S-2, which is attributable to spatial difference in fertilizer broadcasting and consequential input on the soil surface.

Concentration of Cu was higher in S-2 (5.4 ± 1.5 *μ*g g^−1^) followed by S-3 (4.3 ± 0.9 *μ*g g^−1^), S-4 (3.0 ± 0.6 *μ*g g^−1^), S-5 (2.9 ± 0.13 *μ*g g^−1^), and S-1 (1.3 ± 0.4 *μ*g g^−1^) showing the ranking order of S-2 > S-3 > S-4 > S-5 > S-1. Concentration of Cr was higher in S-5 (7.8 ± 0.3 *μ*g g^−1^) followed by S-4 (6.7 ± 0.1 *μ*g g^−1^), S-3 (4.0 ± 0.2 *μ*g g^−1^), S-2 (3.6 ± 0.3 *μ*g g^−1^), and S-1 (2.6 ± 0.1 *μ*g g^−1^) showing the ranking order of S-2 > S-3 > S-4 > S-5> S-1. Concentration of Mn was higher in S-3 (44.2 ± 2.2 *μ*g g^−1^) followed by S-2 (42.1 ± 1.6 *μ*g g^−1^), S-4 (41.2 ± 5.3 *μ*g g^−1^), S-5 (31.5 ± 1.5 *μ*g g^−1^), and S-1 (12.5 ± 5.5 *μ*g g^−1^) showing the ranking order of S-3 > S-2 > S-4 > S-5 > S-1. Concentration of Zn was higher in S-5 (33.8 ± 1.3 ppm) followed by S-4 (28.9 ± 5.5 *μ*g g^−1^), S-2 (17.1 ± 0.9 *μ*g g^−1^), S-3 (14.8 ± 0.4 *μ*g g^−1^), and S-1 (7 ± 0.2 *μ*g g^−1^) showing the ranking order of S-5 > S-4 > S-2 > S-3 > S-1.

The toxic heavy metals Cd and Pb and the micronutrients Zn, Cu, and Mn accumulated in the soil of paddy fields which was higher than that of the control soil. The heavy metals were in a ranking order of Mn > Zn > Pb > Cr > Cu > Cd. The concentrations of Pb, Cd, Cu, Cr, and Zn in the paddy soils are comparable to those for worldwide normal soils (i.e., within the range of published values) [46, 47]. Only concentration of Mn was higher than the value of uncontaminated soil, critical soil concentration [48], and worldwide normal soils (Table 2).

### 3.2. Heavy Metal Concentration in Different Plant Parts

The mean concentrations of heavy metals in the paddy plant parts (Table 1) showed that most of the metals accumulated more in the roots than in other plant parts, shoots, and grains and ranged from 14.4–21.9 *μ*g g^−1^ for Mn, 4.7–16.9 *μ*g g^−1^ for Zn, 3.6–5.3 *μ*g g^−1^ for Pb, 0.6–1.7 *μ*g g^−1^ for Cr, 0.2–0.5 for Cu, and 0.1-0.2 *μ*g g^−1^ for Cd among the five sites (Table 1). It indicated that the Cd concentrations were minimum in the paddy soil, in contrast to the Cd concentrations of paddy soil at Bahour in Puducherry [32]. The mean concentrations of heavy metals in the paddy plant parts showed that most of the metals accumulated more in the roots than in other parts. In general metal uptake was higher for the micronutrients; Mn and Zn in the roots were followed by Pb, Cr, Cu, and Cd. In the present study concentration of Pb was found to be higher in roots that in shoots and grains.* Calluna vulgaris* L. Hull (common heather) and* Agrostis vinealis*, harvested from an abandoned Pb mine in UK, contained 320 and 2930 mg/kg dry wt., respectively, in shoot tissue, while Pb values for root were 9610 and 9740 mg/kg, indicating high plant availability of the Pb in the soil as well as its limited mobility inside the plant [49]. Cu was also found to be more in roots than that in shoots and grains, which is in corroboration with findings of earlier workers [50, 51]. Yang et al. [52] reported that accumulation of Cu was more in roots, while a small fraction (10%) of absorbed Cu was translocated to stem. The Cu and Zn accumulated at their highest concentration in roots of the rice plants followed by shoots and grains. Most metals, Fe, Mn, Zn, and Cu, that were found profusely in the paddy plants were the micronutrients that are required for various enzyme activities and play important roles in photosynthesis and growth of the plant [53, 54].

It was seen that Mn and Cd were accumulated more in shoot than in root and found in the ranges of 25–32.9 *μ*g g^−1^ for Mn, 2.3–6 *μ*g g^−1^ for Zn, 0.4−0.9 *μ*g g^−1^ for Cr, 0.3–1.2 *μ*g g^−1^ for Pb, 0.2-0.3 *μ*g g^−1^ for Cd, and 0.05–0.3 *μ*g g^−1^ for Cu among the five sites (Table 1). In the shoots, concentrations of Mn and Cd were higher than their concentrations in roots and grains. Jarvis, Jones, and Hopper [50] reported that Cd was easily taken up by plants and transported to different parts, although it is nonessential and is of no beneficial effects on plants and animals. Moreover, Cd is toxic to animals and plants, and plants when exposed to this metal show reduction in photosynthesis and uptake of water and nutrient [55]. Higher concentration of Mn in leaves of both the plants indicated its high mobility [56], as leaf chlorophyll content requires Mn for photosynthesis. In contrast, Gupta and Sinha [57] reported higher accumulation of Mn in roots followed by leaves in* Chenopodium*. The mean concentrations of heavy metals in the grains were found in the ranges of 5.6–7.5 *μ*g g^−1^ for Mn, 3.2–7.2 *μ*g g^−1^ for Zn, 0.1–0.6 *μ*g g^−1^ for Cr, 0.1–0.3 *μ*g g^−1^ for Cu, 0.02–0.05 *μ*g g^−1^ for Cd, and 0.01–1 *μ*g g^−1^ for Pb among the five sites (Table 1). In grains, among all metals, Mn and Zn were in more elevated concentrations than Cr, Cu, Cd, and Pb, but their concentrations were less compared to that of roots and shoots. Concentration of Zn ranged between 3.2 *μ*g g^−1^ and 7.2 *μ*g g^−1^, which did not exceed the maximum permissible limit of 50 (Pilc et al.) [58]. Concentration of Pb ranged between 0.01 *μ*g g^−1^ and 1 *μ*g g^−1^. The highest Pb content was found in S-4 (1 *μ*g g^−1^) and S-5 (0.9 *μ*g g^−1^), which exceeds the values given by Pilc et al. [58] or the corresponding limit defined by the Commission Regulation Directive EC [59]. However, the concentrations of Cr, Cu, and Cd ranged between 0.1 *μ*g g^−1^ and 0.6 *μ*g g^−1^, 0.1 *μ*g g^−1^ and 0.2 *μ*g g^−1^, and 0.02 *μ*g g^−1^ and 0.05 *μ*g g^−1^, respectively, which did not exceed the values defined by the Commission Regulation Directive EC [59] or the corresponding limits given by Pilc et al. [58] and FAO/WHO [60] (Table 3). Concentration of Cd was found within the limit defined by CODEX [61] and European Commission [62] (Table 3). Table 3 lists the maximum allowable concentrations (MAC) of Pb, Cd, Cu, Cr, and Zn in foods recommended by the Chinese National Standard Agency. The mean concentrations of all the elements in the rice grain were below their maximum allowable levels except for Pb. The results indicate that the concentration of Pb in rice grain may have been affected by various anthropogenic activities such as use of tractor for farming and use of chemical fertilizers and pesticides. Apart from this, the agricultural watershed is transected on one end by ECR (East Coast Road), a state highway (State Highway number 49) which is used by more than 10,000 vehicles daily. On the other end, Maduvankarai Road, a normal street road connecting the ECR to the boat house via a bridge, also serves as mode of transportation. Both the roads may be contributing to the increase of Pb concentration that may have come from the vehicular emission. Boating, fishing by motor boats, and other recreational activities taking place nearby lagoon could be also a reason for elevated concentration of Pb in rice grain. Concentration of Cr is slightly higher but below MAC. It may be due to some anthropogenic activities such as use of chemical fertilizers and pesticides and other industrial activities near Kalpakkam which comes as runoff and would therefore reflect contamination by the element.

### 3.3. Bioaccumulation Factor

**Figure 2:**
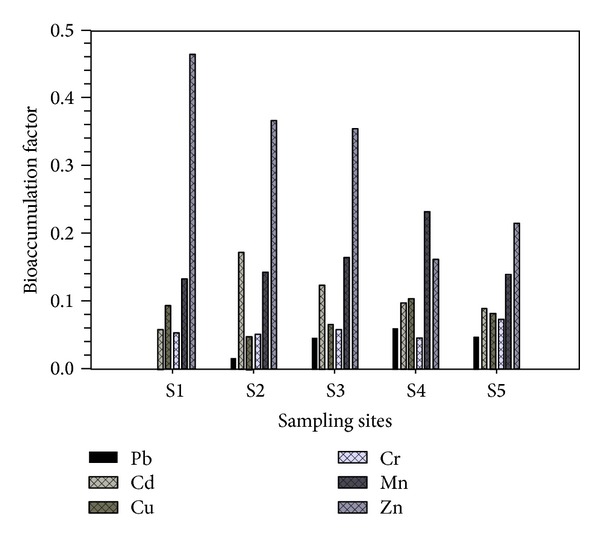
Bioaccumulation factor of the heavy metals across the sites.

Bioaccumulation factors (BAFs) for the heavy metal transfer from soils to rice are shown in Figure 2. The BAF values of the heavy metals such as Zn, Mn, Cd, Cu, Cr, and Pb were found to be in the ranges of 0.2 to 0.5, 0.1 to 0.2, 0.05 to 0.2, 0.04 to 0.1, 0.04 to 0.07, and 0.001 to 0.06, respectively. The trend in the BAF for heavy metals in the study sites was in the ranking order of Zn > Mn > Cd > Cu > Cr > Pb. Among the heavy metals, BAF values were found to be higher for Zn, Mn, and Cd, whereas relatively lower BAF values were found in Cu, Cr, and Pb. The food chain (soil-plant-human) is mainly known as one of the major pathways for exposure of human to soil contaminants. Soil-to-plant transfer is one of the key processes of human exposure to toxic heavy metals through the food chain [63]. When BCF < 1 or BAF = 1, it denotes that the plant only absorbs the heavy metal but does not accumulate when BCF > 1, and this indicates that plant accumulates the heavy metals. BAF values of Pb, Cd, Cu, Cr, Mn, and Zn were less than one in the rice grain which indicates that plants only absorb the heavy metals.

### 3.4. Translocation Factor

Transfer factor is one of the main components of human exposure to toxic heavy metals through the food chain. The transfer factors (TFs) of metals from soil to root (TF_Soil_), root to shoot (TF_Root_), and shoot to grain (TF_Shoot_) were calculated and given in Table 4. The average translocation values of metals in paddy soils from soil to root (TF_Soil_) were found to be in the order of Zn (0.4 to 0.9) > Mn (0.3 to 0.7) > Cd (0.3 to 0.6) > Pb (0.2 to 0.4) > Cr (0.2 to 0.3) > Cu (0.09 to 0.2). In the case of root (root to shoot), TF_Root_ values were found in the order of Cd (1.3 to 2.4) > Mn (1.3 to 2.3) > Cr (0.5 to 0.8) > Cu (0.2 to 0.6) > Zn (0.2 to 0.5) > Pb (0.07 to 0.3). The translocation values for shoot to grain (TF_Shoot_) were found in the following order: Cu (1.1 to 2.5) > Zn (1 to 1.5) > Pb (0.04 to 0.8) > Cr (0.3 to 0.7) > Mn (0.2 to 0.3) > Cd (0.09 to 0.2). There was a significant difference in TFs values among the heavy metals (*P* < 0.05). Soil-to-plant transfer factor is one of the major components of human exposure to metals through the food chain, and it could reveal bioavailability of heavy metals in investigated soils. The higher the TF values are, the more mobile/available the metals are [1, 42, 64]. The TFs vary noticeably within the plant species even for an individual heavy metal [1]. In the present study transfer of Cd and Zn from roots to shoots was more than other heavy metals as the concentrations of these two were found to be more in shoots than that of roots and grains. Roots often contain more Zn than the shoot parts, but the Zn may be translocated from the roots and accumulated in the plant shoot parts [46]. Cd was also translocated more from root to shoot which is known to be relatively mobile in plants [46]. The metal translocation process in plant species is a crucial factor in determining the metal distribution in different plant tissues [65].

### 3.5. Enrichment Factor

**Figure 3:**
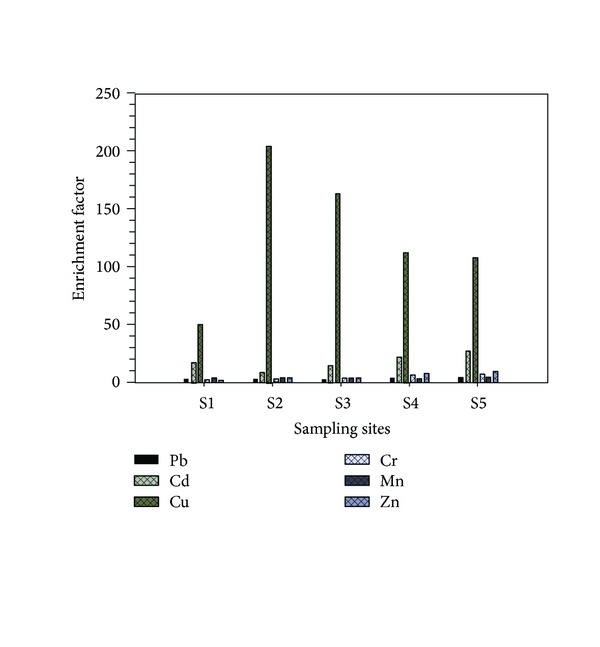
Enrichment factor of the heavy metals in soil across the sites.

The EFs of the paddy field soils for the heavy metals were found to be in the ranges of Pb (2.2 to 3.7), Cd (8.4 to 27.1), Cu (48.6 to 204.3), Cr (2 to 6.2), Mn (3.3 to 4.3), and Zn (1.9 to 9) and in the ranking order of Cu > Cd > Zn > Cr > Mn > Pb. Moreover, there was a significant difference in EFs values among the heavy metals (*P* < 0.01). The EF values greater than 1 indicate higher availability and distribution of metals in the contaminated soil, subsequently increasing the metal accumulation in plants species grown on the soil [36, 37]. Among the metals estimated, the maximal enrichment was found in case of Cu and Cd for the paddy soils (Figure 3).

### 3.6. Correlation Matrix

The correlation coefficient matrix is normally used to measure the degree of correlation between logarithms of the elemental concentrations [70], and this matrix for the heavy metals of paddy showed highly significant positive correlations between the pairs of elements of soil samples—Cd-Pb (0.65), Cr-Pb (0.95), Zn-Pb (0.96), Cd-Cr (0.83), Cd-Zn (0.69), and Cr-Zn (0.98) (Table 5(a)). Besides, highly significant positive correlations were found between the pairs of elements present in grains which are Pb-Cd (0.92), Pb-Cu (0.75), Cr-Pb (0.73), Mn-Pb (0.96), Cd-Cr (0.91), Mn-Cd (0.98), Zn-Cd (0.71), Cr-Mn (0.85), and Cr-Zn (0.72) (Table 5(b)). The significant positive correlation between the elements of surface soil grains suggests that their common source of origin is probably the agrochemicals, such as phosphate and nitrate fertilizers, broadcasted in the paddy fields [32].

### 3.7. Potential Health Risk of Heavy Metals through Rice Intake

Rice consumption has been identified as one of the major pathways of human exposure to the toxic heavy metals accumulated in rice grain.  Table 6 showed the dietary intake (DI) of heavy metals via rice for adults and children in the study region as the local people consume generally rice, the staple food for the people available in the region. The DIs of Pb, Cd, Cu, Cr, and Zn through rice were estimated to be 4.02, 0.27, 1.66, 1.98, and 37.04 mg/kg/day for adults and 3.50, 0.23, 1.45, 1.73, and 32.28 mg/kg/day for children, respectively. The DIs of heavy metals for adults were found to be higher than those for children. This is most probably due to relatively higher quantity of rice consumption of adults compared to the children, which increased the DIs of heavy metals. The result is in conformity with previous studies in the neighboring country, China [40, 71].

The HRIs of heavy metals through rice consumption are given in Table 4. The HRI of heavy metals for adults from rice consumption was in decreasing order: Zn > Pb > Cr > Cd > Cu. The HRI of heavy metals for children also has the similar trend as the adults. The Zn has the highest HRI value as it is an essential micronutrient. Hence, it may not pose a potential risk up to a certain concentration but may cause adverse effects at certain elevated level. So among the toxic heavy metals, Pb ingestion has the highest potential health risk of adverse effects for adults and children and Cu ingestion has minimum risk. Only HRI of Zn for adults was found beyond the value of 1. Therefore, the health risk of single metal exposure through rice consumption was generally assumed to be safe for the people of the region. However, local inhabitants may be at risk due to the combination of several toxic heavy metals [71]. The HI values for rice consumption of adults and children were 1.561 and 1.360, respectively. This indicates that adults and children may experience poor health effects in the near future as the heavy metal accumulation over a period of time leads to biomagnification. Our assessment was only to measure the intake of toxic heavy metals through rice consumption. In fact, humans are also exposed to heavy metals through other foods/pathways such as consumption of contaminated vegetables, fruits, fish, meat, water, and milk [40, 41, 72, 73]. Moreover, there may be the other sources such as dust inhalation and dermal contact [74, 75].

## 4. Conclusion

The present study carried out on paddy fields near Kalpakkam in Tamil Nadu, South India, determined the accumulation of essential and nonessential heavy metals in paddy soils as well as in rice plants including paddy grains. Theoccurrence of heavy metals in paddy field soils was in a ranking order of Mn > Zn > Pb > Cr > Cu > Cd. Concentrations of the heavy metals were higher in paddy field soils compared with the control soil. However, the concentrations of Pb, Cd, Cu, Cr, and Zn except for Mn in the paddy soils were comparable to those of worldwide normal soils, which were higher than the value of uncontaminated soil. The uptake of Mn and Zn was higher in the roots of paddy plants, which were followed by Pb, Cr, Cu, and Cd. Mn and Cd accumulated more in the shoots than in roots and grains. Pb content in grains exceeded the maximum permissible value in S-4 and all other metals were below the safe limits. Estimations showed that DIs of heavy metals for adults were found to be higher than those for children, which was most probably due to relatively higher quantity intake by adults. In general, no HRI values were >1 through rice consumption except for Zn for adults. The HRI values for both adults and children were 1.561 and 1.360, respectively, indicating that both adults and children may experience some adverse health effects in the future, since chemical fertilizers and pesticides are indiscriminately used by Indian farmers, which are probably the main sources of the toxic heavy metals accumulated in the paddy fields. Organic agriculture with little use of agrochemicals could be the alternative solution for reducing the contamination of toxic heavy metals particularly the toxic Cd, Cr, and Pb in the paddy fields producing rice, the staple food in India and other Southeast Asian countries.

## Figures and Tables

**Table 1:** Mean concentrations of heavy metals along with standard deviation in soil and different plant parts across the sampling sites.

Heavy metals	Soil range	Root range	Shoot range	Grain range
	(*μ*g g^−1^)	(*μ*g g^−1^)	(*μ*g g^−1^)	(*μ*g g^−1^)
Pb	5.3 ± 0.4–19.8 ± 1.3	3.6 ± 0.2–5.3 ± 0.04	0.3 ± 0.01–1.2 ± 0.01	0.01 ± 0.001–1 ± 0.02
Cd	0.02 ± 0.005–0.6 ± 0.04	0.11 ± 0.008–0.2 ± 0.01	0.2 ± 0.01–0.3 ± 0.01	0.02 ± 0.001–0.05 ± 0.002
Cu	0.03 ± 0.004–5.4 ± 1.5	0.2 ± 0.02–0.5 ± 0.04	0.04 ± 0.008–0.3 ± 0.03	0.1 ± 0.008–0.3 ± 0.01
Cr	1.3 ± 0.01–7.8 ± 0.3	0.6 ± 0.02–1.7 ± 0.04	0.4 ± 0.01–0.9 ± 0.04	0.1 ± 0.02–0.6 ± 0.01
Mn	12.5 ± 0.2–53.9 ± 1.5	14.4 ± 0.9–21.9 ± 0.3	25 ± 2.8–32.9 ± 1.9	5.6 ± 0.04–7.5 ± 0.03
Zn	3.8 ± 1.7–33.8 ± 1.3	4.7 ± 0.1–16.9 ± 0.9	2.3 ± 0.01–6 ± 0.2	3.2 ± 0.05–7.2 ± 0.008

**Table 2:** Mean values of heavy metals (Pb, Cd, Cu, Cr, Mn, and Zn) for uncontaminated paddy soils, mean values for worldwide normal surface soils, critical concentrations for contaminated soils, Indian standards, and European Union standards, compared with the values of present study.

Elements (*μ*g g^−1^)	Mean values for paddy soils^a^	Mean values for worldwide normal surface soils ^b^	Critical soil concentration^c^	Indian standards^d^	European Union standards^e^ (EU 2002) [69]	Present study
Pb	23.3	22–44	100–400	250–500	300	5.3–19.8
Cd	0.34	0.37–0.78	3–8	3–6	3	0.2–0.6
Cu	20.7	13–24	60–125	135–270	140	1.10–2.9
Cr	64	12–83	75–100	—	—	1.3–7.8
Mn	0.39	0.27–0.53	1.5–3	—	—	12.5–33.9
Zn	61	45–100	70–400	300–600	300	3.8–33.8

^a^Mean of total concentrations of elements in uncontaminated paddy soils. Data are from Wong et al. (2002) [4]; Wang et al. (2003) [66];    Chandrajith et al. (2005) [67].

^b^Mean of total concentrations of elements in the surface horizon of worldwide normal soil, and normal means that the data do not include contaminated or mineralized soils. Data are from Kabata-Pendias (2001) [46]; Essington (2004) [68].

^c^Higher concentrations may be toxic to plants depending on speciation (Alloway, 1995) [48].

^d^Indian standards (Awashthi, 2000) [47] for agricultural soils.

^e^European standards (EU 2002) [69] for agricultural soils.

**Table 3:** The total concentrations of Pb, Cd, Cu, Cr, Mn, and Zn in rice (*Oryza sativa* L.) samples from the experimental sites compared with the standard values.

Elements (*μ*g g^−1^)	1	2	3	4	5	6	Present study
Pb	0.2	—	1.0	—	—	0.2	0.01–1.0
Cd	0.1	—	0.15	0.4	0.2	0.2	0.02–0.05
Cu	—	10	—	—	—	10	0.1–0.23
Cr	—	—	—	—	—	1.0	0.13–0.56
Mn	—	—	—	—	—	—	5.58–7.47
Zn	—	—	50	—	—	50	3.23–7.24

Maximum permitted levels for heavy metals in food crops: (1) Commission Regulation Directive EC (2001) [59]; (2) FAO/WHO (1992) [60]; (3) Pilc et al. (1994) [58]; (4) CODEX Alimentarius Commission (2006) [61] and (5) European Food Safety Authority, EFSA [62].

(6) maximum levels of contaminants in foods (GB 13106-1991; GB 2762-2005; GB 15199-1994; GB 4810-1994) [7].

**Table 4:** Ranges of the translocation factors of the heavy metals from soil to root, root to shoot, and shoot to grain across the sampling sites.

Heavy metals	Tf (soil to root)	Tf (root to shoot)	Tf (shoot to grain)
Pb	0.2–0.4	0.1–0.3	0.04–0.8
Cd	0.2–0.6	1.4–2.4	0.1-0.2
Cu	0.1-0.2	0.2–0.6	1.1–2.5
Cr	0.2-0.3	0.5–0.8	0.3–0.7
Mn	0.3–0.7	1.3–2.3	0.2-0.3
Zn	0.4–0.9	0.3–0.5	1–1.5

**Table:** (a)

	Pb	Cd	Cu	Cr	Mn	Zn
Pb	1					
Cd	0.654123	1				
Cu	0.193626	−0.61182	1			
Cr	0.952659	0.802794	−0.06512	1		
Mn	0.316071	0.273936	0.032893	0.175871	1	
Zn	0.967693	0.699247	0.079194	0.984368	0.192237	1

**Table:** (b)

	Pb	Cd	Cu	Cr	Mn	Zn
Pb	1					
Cd	0.924811	1				
Cu	0.759248	0.629072	1			
Cr	0.733659	0.915057	0.267458	1		
Mn	0.968289	0.988837	0.716005	0.850878	1	
Zn	0.447148	0.719954	0.42386	0.722836	0.64716	1

**Table 6:** Health risk assessment of heavy metals via intake of rice.

Individuals	Element	ORD	DI	HRI	HI
Adults	Pb	3.50	4.02	0.269	1.561
	Cd	1.00	0.27	0.042	
	Cu	40.00	1.66	0.001	
	Cr	1500.00	1.98	0.123	
	Zn	300.00	37.04	1.126	

Children	Pb	3.50	3.50	0.234	1.360
	Cd	1.00	0.23	0.036	
	Cu	40.00	1.45	0.001	
	Cr	1500.00	1.73	0.108	
	Zn	300.00	32.28	0.981	

ORD oral reference dose (in micrograms per kilogram per day), DI daily intake (in micrograms per kilogram per day).
